# Multiplexed protein profiling by sequential affinity capture

**DOI:** 10.1002/pmic.201500398

**Published:** 2016-03-31

**Authors:** Burcu Ayoglu, Elin Birgersson, Anja Mezger, Mats Nilsson, Mathias Uhlén, Peter Nilsson, Jochen M. Schwenk

**Affiliations:** ^1^ Affinity ProteomicsSciLifeLabSchool of BiotechnologyKTH ‐ Royal Institute of TechnologySolnaSweden; ^2^ Department of Biochemistry and BiophysicsSciLifeLabStockholm UniversitySolnaSweden

**Keywords:** Affinity proteomics, Antibody arrays, Plasma profiling, Suspension bead arrays

## Abstract

Antibody microarrays enable parallelized and miniaturized analysis of clinical samples, and have proven to provide novel insights for the analysis of different proteomes. However, there are concerns that the performance of such direct labeling and single antibody assays are prone to off‐target binding due to the sample context. To improve selectivity and sensitivity while maintaining the possibility to conduct multiplexed protein profiling, we developed a multiplexed and semi‐automated sequential capture assay. This novel bead‐based procedure encompasses a first antigen capture, labeling of captured protein targets on magnetic particles, combinatorial target elution and a read‐out by a secondary capture bead array. We demonstrate in a proof‐of‐concept setting that target detection via two sequential affinity interactions reduced off‐target contribution, while lowered background and noise levels, improved correlation to clinical values compared to single binder assays. We also compared sensitivity levels with single binder and classical sandwich assays, explored the possibility for DNA‐based signal amplification, and demonstrate the applicability of the dual capture bead‐based antibody microarray for biomarker analysis. Hence, the described concept enhances the possibilities for antibody array assays to be utilized for protein profiling in body fluids and beyond.

**Abbreviations**
DCA: dual capture assay
IGFBP2: insulin‐like growth factor binding protein 2
MFI: median fluorescence intensity
PSA: prostate specific antigen
RCA: rolling circle amplification
SAPE: R‐phycoerythrin conjugated streptavidin

Albeit great advances in proteomics with mass spectrometry (MS), there is still a challenge when it comes to the analysis of body fluids such as blood plasma due to complex sample composition or available quantities. To address this, there are ongoing efforts to provide affinity reagents to all human proteins and to use these across technology platforms and a variety of specimen 1. Among these technologies bound for proteomic analyses, antibody arrays hold the promise to conduct advanced protein profiling in body fluid samples such as serum, plasma, cerebrospinal fluid or urine 2.

The discovery‐driven antibody array format uses single antibody assays for highly parallelized multiplex analysis and offers a very efficient strategy for antibody‐based discoveries in large sample collections 3, 4, 5. Single antibody assays consume little amount of reagents and samples but do not match sensitivity and selectivity of preferentially used sandwich immunoassays (Supporting Information Fig. S1) due to increased background noise and off‐target interference of higher abundant proteins 6. The latter type dual binder assays may though be often limited in flexibility to accommodate and combine assays for many targets as compared to single binder assays with labeled samples 7. Analysis via directly labeled specimen and single antibody interactions remains therefore attractive as a discovery tool, even though such assays may suffer from off‐target binding to proteins of higher abundance or epitope similarity. Furthermore, certainty about the validity of the single binder assay data cannot be judged before additional assays using the same sample material and enrichment principles, such as sandwich immunoassays 8 or MS readout 9, are applied.

In order to advance current single binder assay designs, off‐target binding must be reduced to improve assay selectivity and ultimately sensitivity. To address this, we developed an assay concept, which is based on two sequential affinity binding events using two complementary single binder arrays. The principle of this dual capture assay (DCA) is illustrated in Fig. 1. A similar approach was previously described for single analyte assays utilizing microtiter plates as solid support 10. Our proof‐of‐concept study expands on such efforts and utilizes the flexibility offered by magnetic beads as solid support for multiplexed enrichment and read‐out, as well as a combinatorial high and low pH elution. The presented work was built on more than 50 antibodies that are parts of commercially available sandwich assay kits in order to allow comparisons between sandwich, dual‐ and single‐binder assays (Supporting Information Table S1). These sandwich assay antibodies were not validated for any single or DCA use previously.

**Figure 1:**
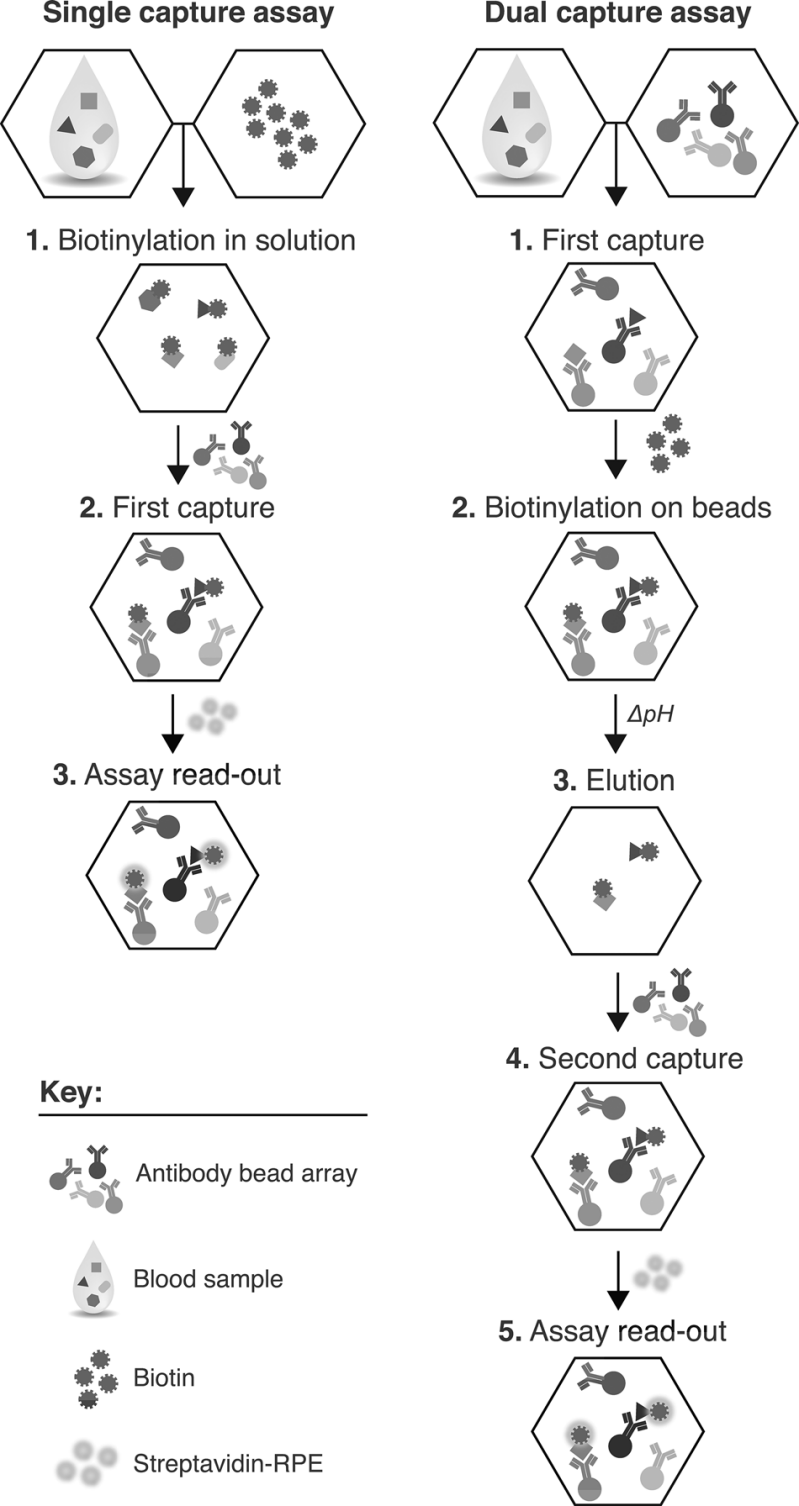
The concept of the dual capture assay (DCA). For the first affinity capture, a neat sample is incubated with the first set of antibody‐coupled beads. The beads are subsequently washed to remove unbound proteins and subjected to labeling with biotin. Captured and labeled target proteins are then eluted first by high pH, followed by low pH treatment, both in a small sample volume. The two eluates are then neutralized to pH 7 and a second set of beads is added to capture and detect the remaining target proteins and analyze the bead array in a flow‐cytometer. In comparison to this DCA, single capture assays rely on direct capture of targets biotinylated in solution and subjected to read‐out. (For simplicity, an ideal assay with no off‐target interactions is illustrated.)

The DCA protocol is conducted as follows: for the first affinity capture, a neat sample is incubated with the first antibody bead array; beads are then washed and subjected to labeling at a 300‐fold molar access of biotin. Thus, captured proteins and the immobilized antibodies are modified, available for later read‐out and also QC purposes that determine if captured proteins (and antibodies on beads) have been labeled accordingly. Several conditions such as the effect of detergent during biotin‐labeling step (Supporting Information Fig. S2) and pH levels for releasing antibody‐bound proteins were evaluated (Supporting Information Figs. S3 and S4). This led to subsequent release of proteins in a series of first high and then low pH elution in a small sample volume (15 μL), followed by heat treatment (56°C). The two eluates were combined and neutralized at pH 7 upon mixing in a TRIS‐based assay buffer. For detection, a second bead array is used to capture those labeled target proteins remaining in the neutralized eluate. Hence, a less complex sample environment has been created for read‐out analysis by enriching proteins from a neat serum or plasma sample, thus reducing present quantities of both on‐ and off‐target and allowing for an improved assay selectivity (Fig. 2A–B, Supporting Information Fig. S5).

**Figure 2:**
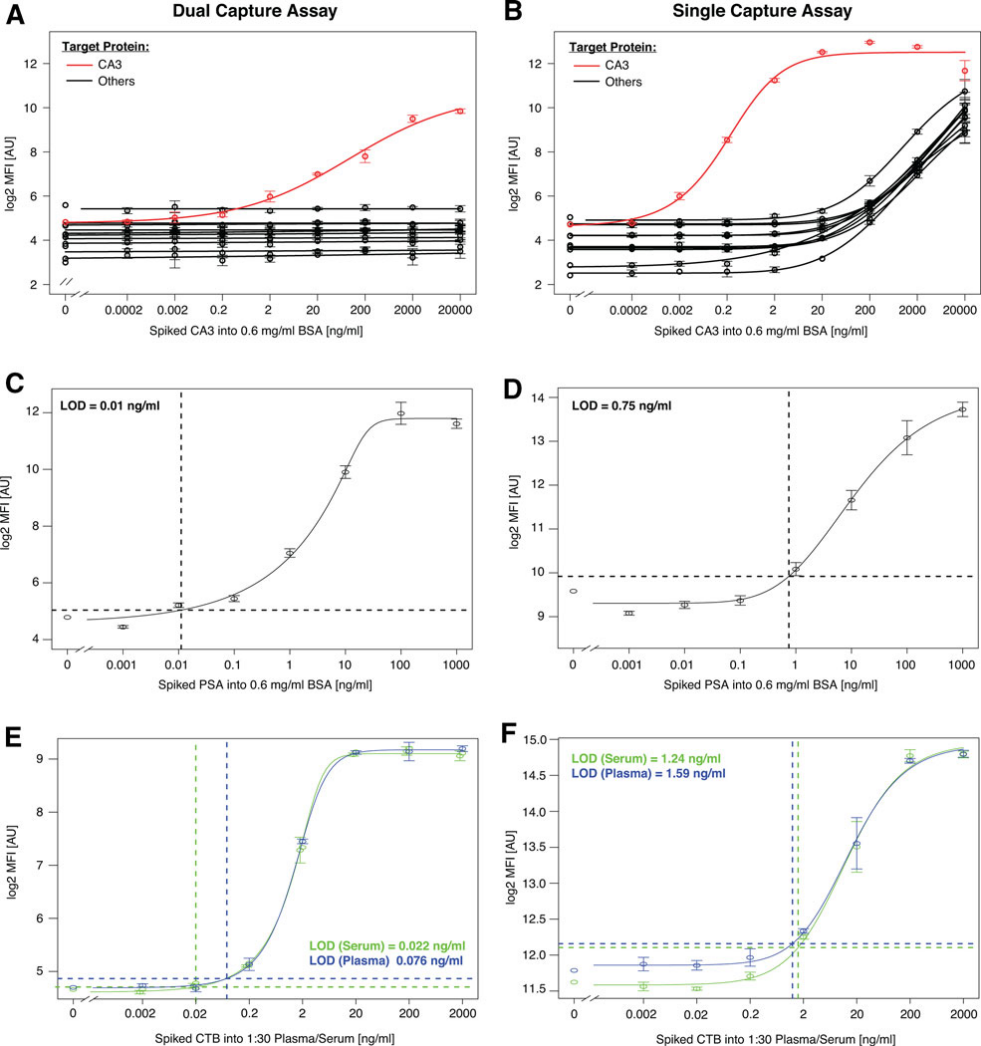
Performance comparison on selectivity and sensitivity. (A–B) A ten‐fold dilution series of carbonic anhydrase III protein spiked into 0.6 mg/mL BSA was analyzed with a 52‐plex antibody array (Supporting Information Table S1) both in a single‐ and dual‐capture assay format. The y‐axes display the MFI values obtained for a subset of the antibodies including the anti‐carbonic anhydrase III antibody (red). (C–D) Similarly, a ten‐fold dilution series of PSA was spiked into 0.6 mg/mL BSA and analyzed with a 52‐plex antibody array both in a single‐ and dual‐capture assay format. The y‐axes display the MFI values obtained for the anti‐PSA antibody. (E–F) The bacterial protein cholera toxin subunit B was spiked into a 1:30 diluted human plasma or serum sample and analyzed with a 52‐plex antibody array both in a single‐ and dual‐capture assay format. The y‐axes display the MFI values obtained for the anti‐cholera toxin subunit B antibody. In each subfigure, the x‐axes display concentrations of the investigated proteins in ng/mL. All measurements were performed in triplicates. Error bars indicate SD. LODs were calculated using a 5‐parametric model and the obtained levels for each analysis are indicated as insets.

The DCA concept was further tested in various constellations. At first, we evaluated how the number of beads used in first capture influenced the degree of assay selectivity (Supporting Information Fig. S6). We then tested the use of only a single antibody during first capture to assess the degree of off‐target interaction and the potential to measure released proteins by the secondary array (Fig. 3F). Next, the sensitivity of the assay was compared to both single capture (Fig. 2, Supporting Information Fig. S1) as well as to sandwich assays (Supporting Information Fig. S1). When compared to single capture assays, the background observed by antibodies to which no target had been spiked into the sample was low (median fluorescence intensity, MFI < 50) over several sample concentrations and generally not affected by sample complexity (Fig. 2A–B, Supporting Information Fig. S5).

**Figure 3:**
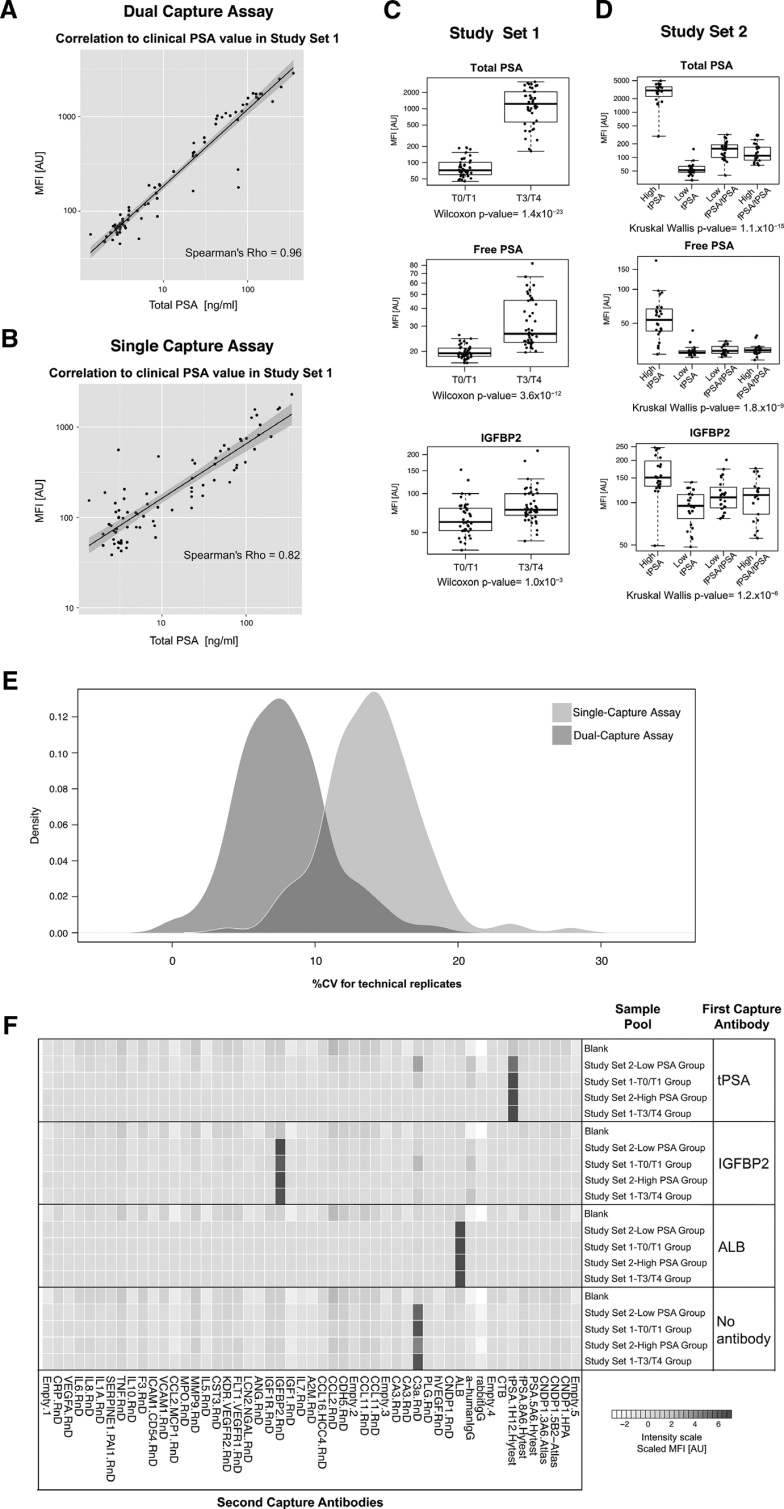
Selectivity and biomarker analysis in prostate cancer. Two different prostate cancer plasma sample sets, denoted as Study Set 1 and Study 2 as described in Supporting Information Table S1, were analyzed using a 52‐plex antibody array. (A–B) For Study Set 1, we compared PSA values determined in the clinic with data generated in both a single‐ and dual‐capture assay format. The y‐axes in the scatterplots display the MFI values for the anti‐total PSA antibody and the x‐axes display the total PSA concentration determined in the clinic. In these scatterplots, MFI values for only those samples with clinical PSA value < 1 μg/mL are shown. Scatterplots in Supporting Information Fig. S9 display all samples including those with PSA > 1 μg/ml. (C–D) A univariate analysis of the protein profiles obtained within Study Set 1 and Study Set 2 using the dual capture assay revealed the most prominent differences for three antibodies targeting total PSA, free PSA and IGFBP2. The boxplots display the MFI values for each sample categorized into the T0/T1 or T3/T4 group within Study Set 1 and into four different groups pre‐determined based on clinical total and free PSA values within Study Set 2. A more detailed overview of the differences revealed for all antibodies included in the array is provided in Supporting Information Table S3. (E) Six replicates of a sample‐free blank and four replicates of a plasma sample pool were included to assess the intra‐assay CV in the analysis of Study Set 1 using both a single‐ and a dual‐capture assay format. The density plot displays the distribution of percentage of CV across all antibodies in the technical replicates. (F) Instead of using all 52 antibodies combined for first capture enrichment, single bead populations either coupled to an anti‐total PSA, anti‐IGFBP2, anti‐ALB or without any antibody were used for first capture. The heatmap displays the scaled MFI values obtained for pools of the different sample groups within Study Set 1 and 2 when only a single antibody or no antibody was used in first capture.

As described above, DCA showed improved selectivity and low background levels. To make use of the observations and to enhance sensitivity, we applied and evaluated an on‐bead signal amplification protocol using rolling‐circle amplification for multiplexed protein detection (Supporting Information Fig. S7). As shown in Supporting Information Fig. S8, there was a high concordance between R‐phycoerythrin conjugated streptavidin (SAPE) and rolling circle amplification (RCA)‐based readout (Pearson's *r* > 0.9). The average CV obtained from triplicates of a plasma dilution series was slightly higher with RCA detection (CV = 9.8%) compared to SAPE (CV = 4.6%) (Supporting Information Fig. S9), yet the RCA derived background levels (MFI = 9) were lower than for SAPE readout (MFI = 15) when assessing this using beads that were prepared using an antibody‐free coupling solution. This proof‐of‐concept test demonstrated the feasibility of using RCA on beads for DCA analysis but follow‐up studies would be needed to further assess the performance characteristics of RCA by using more antibodies.

Finally, we challenged the DCA concept in a biomarker analysis setup by investigating two different plasma sample sets collected in the context of prostate cancer (Supporting Information Table S2) 8, 11, 12. Using multiple antibodies for multiplexed first capture enrichment, we found differential profiles primarily for prostate specific antigen (PSA, as expected) as well as for insulin‐like growth factor binding protein 2 (IGFBP2, Fig. 3C–D). Subsequently, we confirmed on‐target detection using only anti‐PSA or anti‐IGFBP2 antibody beads during first capture enrichment and subsequent multiplexed detection (Fig. 3F). Compared to previously used single binder assay analysis 11, the correlation between clinically determined PSA and DCA‐derived levels was very high (Rho > 0.95) and in a linear relation to clinical PSA values < 1 μg/mL (Fig. 3A–B). At higher PSA values, the DCA assay reached saturation levels (Supporting Information Fig. S10) and we speculate that using more beads in the first capture step could expand the dynamic range to beyond 1 μg/mL. In the current setting and using a 5‐parametic fitting function, we estimated a limit of detection for PSA using the DCA assay to 11 pg/mL, thus improving assays sensitivity to direct labeling single capture assays by almost 70‐fold (Fig. 2E–F). Compared to single binder assays, we also found that the correlation between random pairs of antibody profiles was reduced in DCA (Supporting Information Fig. S11). While both approaches still benefit from data normalization, the sample dependent background was less influential in DCA, because many more antibodies revealed signals of low intensity levels. We hypothesize that this was reflected by the lowered correlation of random antibody pairs. On top of this, the technical variability for DCA (CV ± 5%) was lower than for single binder assays (%CV ± 15%) (Fig. 3E). Besides these technical aspects, the p‐values determined in each of the two different prostate cancer sample sets were computed for single capture and DCA. This revealed that analysis of DCA data generated lower p‐values for PSA and suggested a smaller number of tentative candidates (Supporting Information Table S3). We and others have acknowledged that antibody performance is application dependent 13. In this setting, we believe that the observed differences from single binder assay may still be valid if confirmed by other assays, for example the presented DCA concept. As shown in Fig. 3F, the use of a single antibody for first capture allows to assess selectivity of the enrichment in relation to the composition of the second capture array. While anti‐PSA, anti‐IGFBP2 and anti‐ALB revealed an on‐target enrichment, eluates from a bead carrying no antibody contained proteins that were recognized by an anti‐C3a antibody. The latter, presumably off‐target detection, would call for further investigations either to confirm whether C3a is enriched due to interactions with antibody‐free beads or whether anti‐C3a binds to another protein than C3a.

The use of antibody coupled beads for sandwich immunoassay 14 thus presumably also the direct labeling‐based antibody arrays follow the conditions of ambient analyte analysis 15. This implies that the measured intensity levels are dependent on target concentration rather than overall available quantities (mass sensing). At equal concentration, it is therefore likely that on‐target interactions are preferred over off‐target due to stronger affinities between antibody and target protein. Thus, an environment for a more selective assay can be achieved in DCA's second round of affinity capture, where both on‐ and off‐target levels are expected to be lower than in the neat sample. Even though DCA does not require accommodating two binders on one protein at the same time, a possible limitation of this concept can arise when antibodies reveal affinities that are similar for off‐target and on‐target. For such scenarios, complementarity between first and second capture array has to involve additional binders that then bind to different regions of the proteins. The DCA concept even offers to accommodate different types of affinity scaffolds, such as affibody molecules 16, aptamers 17, or DARPins 18, for one of the two capture steps. This could address sample components that bind to common regions of those affinity reagents used during first and second capture. A major advantage of the DCA concept is the on‐bead labeling step, as this allows for removal of buffers that may otherwise be incompatible with NHS esters used for biotin‐labeling (e.g. TRIS‐HCl) and for avoiding additional steps for sample pre‐processing. This feature could open up possibilities for multiplex protein profiling in other sample types such as extracts of tissues and cells. Another option for DCA that can be further explored is the use of different additives and detergents to stabilize or denature targets. Since two incubation steps are used, proteins are currently assumed to be in a native‐like form during the first enrichment step, while the read‐out is achieved based on denatured proteins. Lastly, labeling an affinity‐captured protein may be beneficial and allow for protecting the epitope from the labeling agent. Consequently, capturing such a protein again could be more efficient as compared to capturing a protein labeled in solution.

In summary, the developed sequential affinity capture assay facilitates multiplexed analysis of proteins in plasma. In the proof‐of‐concept study, it showed improved performance characteristics compared to classical antibody arrays thus may serve as a first alternative when developing sandwich assays with antibodies found indicative in single binder screening efforts. The results from DCA experiments were in good agreement with clinical analysis. This miniaturized and parallelized concept holds promise to further advance antibody‐based discoveries of soluble biomarker molecules in body fluids, and with suitable magnetic bead handling and automation, is ready for larger study sets.

*The authors have declared no conflict of interest*.

## Supporting information

As a service to our authors and readers, this journal provides supporting information supplied by the authors. Such materials are peer reviewed and may be re‐organized for online delivery, but are not copy‐edited or typeset. Technical support issues arising from supporting information (other than missing files) should be addressed to the authors.

Supplementary Figure S1. Comparison of LOD values in DCA, single‐capture assay and sandwich assay
Supplementary Figure S2. Effect of detergent added during the labeling step on limit of detection in DCA
Supplementary Figure S3. Effect of high or low pH during elution on limit of detection in DCA
Supplementary Figure S4. Comparison of pH conditions during elution on overall protein profiles in plasma
Supplementary Figure S5. Selectivity in DCA compared to single capture assay
Supplementary Figure S6. Effect of number of beads at first capture
Supplementary Figure S7. Assay read‐out with rolling circle amplification
Supplementary Figure S8. Effect of RCA on overall MFI values
Supplementary Figure S9. CVs in SAPE‐ and RCA‐based readout
Supplementary Figure S10. Correlation to clinical PSA values in Study Set 1 and 2
Supplementary Figure S11. Distribution of antibody correlation coefficients in DCA and single‐capture assay
Supplementary Table S1. Information on the antibodies used in the study
Supplementary Table S2. Information on the two prostate cancer plasma sample collections
Supplementary Table S3. P‐values for differences revealed in two different prostate cancer study sets
Supplementary Table S4. Sequence of the oligonucleotides used in RCA
