# Supplementary material for: Multiplexed protein profiling by sequential affinity capture
Source: Proteomics. 2016 Mar 31;16(8):1251–6. doi: 10.1002/pmic.201500398 (PMC5071697; doi:10.1002/pmic.201500398)
Supplement: Supplementary file 1 — Supplementary Figure S1. Comparison of LOD values in DCA, single‐capture assay and sandwich assay Supplementary Figure S2. Effect of detergent added during the labeling step on limit of detection in DCA Supplementary Figure S3. Effect of high or low pH during elution on limit of detection in DCA Supplementary Figure S4. Comparison of pH conditions during elution on overall protein profiles in plasma Supplementary Figure S5. Selectivity in DCA compared to single capture assay Supplementary Figure S6. Effect of number of beads at first capture Supplementary Figure S7. Assay read‐out with rolling circle amplification Supplementary Figure S8. Effect of RCA on overall MFI values Supplementary Figure S9. CVs in SAPE‐ and RCA‐based readout Supplementary Figure S10. Correlation to clinical PSA values in Study Set 1 and 2 Supplementary Figure S11. Distribution of antibody correlation coefficients in DCA and single‐capture assay Supplementary Table S1. Information on the antibodies used in the study Supplementary Table S2. Information on the two prostate cancer plasma sample collections Supplementary Table S3. P‐values for differences revealed in two different prostate cancer study sets Supplementary Table S4. Sequence of the oligonucleotides used in RCA [file PMIC-16-1251-s001.docx]

**SUPPLEMENTARY INFORMATION**

**Multiplexed protein profiling by sequential affinity capture**

*Burcu Ayoglu* ^1)^*, Elin Birgersson* ^1)^*, Anja Mezger* ^2)^*, Mats Nilsson* ^2)^*, Mathias Uhlén* ^1)^*, Peter Nilsson* ^1)^*, and Jochen M. Schwenk* ^1)^

^1)^ Affinity Proteomics, SciLifeLab, School of Biotechnology, KTH - Royal Institute of Technology, Box 1031, SE-171 21 Solna, Sweden

^2)^ Department of Biochemistry and Biophysics, SciLifeLab, Stockholm University, Box 1031, SE-171 21 Solna, Sweden

**INDEX**

Abbreviations 2

Materials and Methods 3

Protocol for generation of the antibody bead arrays 3

Protocols for biomarker discovery analysis in prostate cancer 4

Prostate cancer plasma samples 4

Direct labeling and single-capture assay of prostate cancer samples 4

Dual-capture assay of prostate cancer samples 5

Protocols for spike-in experiments with protein standards and plasma dilution series 6

Direct labeling and single-capture assay with protein standards or plasma dilution series 6

Dual capture assay with protein standards or plasma dilution series 6

Protocol for sandwich assay on protein standards 6

Protocol for assay read-out by RCA 7

Streptavidin oligonucleotide conjugation 7

Padlock probe phosphorylation 7

Signal amplification by RCA 8

Data analysis and statistics 8

Supplementary Figures & Tables 9

Supplementary Fig. 1 – Comparison of LOD values in DCA, single-capture assay and sandwich assay 9

Supplementary Fig. 2 – Effect of detergent added during the labeling step on limit of detection in DCA 10

Supplementary Fig. 3 – Effect of high or low pH during elution on limit of detection in DCA 11

Supplementary Fig. 4 – Comparison of pH conditions during elution on overall protein profiles in plasma 13

Supplementary Fig. 5 – Selectivity in DCA compared to single capture assay 14

Supplementary Fig. 6 – Effect of number of beads at first capture 15

Supplementary Fig. 7 – Assay read-out with rolling circle amplification 16

Supplementary Fig. 8 – Effect of RCA on overall MFI values 18

Supplementary Fig. 9 – CVs in SAPE- and RCA-based readout. 19

Supplementary Fig. 10 – Correlation to clinical PSA values in Study Set 1 and 2 20

Supplementary Fig. 11 – Distribution of antibody correlation coefficients in DCA and single-capture assay 21

Supplementary Tab. 1 – Information on the antibodies used in the study 22

Supplementary Tab. 2 – Information on the two prostate cancer plasma sample collections 23

Supplementary Tab. 3 – P-values for differences revealed in two different prostate cancer study sets 24

Supplementary Tab. 4 – Sequence of the oligonucleotides used in RCA 25

References 26

# Abbreviations

BSA – bovine serum albumin; CA3 – carbonic anhydrase III; CTB – cholera toxin subunit B; DCA – dual capture assay; IGFBP2 – insulin-like growth factor binding protein 2; LOD – limit of detection; MFI – median fluorescence intensity; PSA – prostate specific antigen; RCA – Rolling circle amplification; R-PE – R-Phycoerythrin; SAPE – R-Phycoerythrin conjugated streptavidin; SD – standard deviation; SOTA – Self-organizing tree algorithm

# Materials and Methods

### Protocol for generation of the antibody bead arrays

The concentration of the utilized antibodies was normalized by diluting 3.2 μg of each antibody into 100 μl of 0.1 M 2-(N-morpholino)ethanesulfonic acid (MES) buffer (pH 4.5). Antibodies were then coupled to carboxylated, color-coded magnetic beads (MagPlex-C, Luminex Corp.) as per previously described antibody-coupling protocols [1]. In brief, 1x10^6^ beads per bead identity were distributed across 96-well microtiter plates (Greiner BioOne), washed and re-suspended in phosphate buffer (0.1 M NaH_2_PO_4_, pH 6.2) using a plate washer (EL406, Biotek). Bead surfaces were activated by addition of 0.5 mg 1-ethyl-3(3-dimethylamino-propyl)carbodiimide (Pierce) and 0.5 mg N-hydroxysuccinimide (Pierce) in 100 μl phosphate buffer. After 20 min incubation on a shaker (Grant Bio), beads were washed with 0.1 M MES buffer. Pre-diluted antibodies were added to the beads and incubated for 2 h at RT. In all coupling rounds, additional bead identities were functionalized either with 3.2 μg of rabbit IgG (Bethyl), 3.2 μg of recombinant human albumin (Dako), or without addition of any protein providing assay quality controls. Antibody-coupled beads were then washed 3x in PBS-T (1×PBS, 0.05% Tween20), re-suspended in 50 μl of a storage buffer (Blocking Reagent for ELISA, Roche Applied Science) supplemented with 0.1% (v/v) ProClin (Sigma-Aldrich) and stored overnight at 4°C. In order to generate the 52-plex bead array with the full set of antibodies listed in Table S1, equal volumes of each bead identity were combined. For the experiments with single antibody capture, bead populations coupled to only one antibody were used. Before use, the bead mixtures were sonicated for 3 min (Branson Ultrasonic Corp.). The bead arrays were stored at 4°C until further use. The coupling of each antibody on the beads was confirmed via R-Phycoerythrin (R-PE)-conjugated secondary reagents, such as R-PE conjugated donkey anti-rabbit IgG antibody (Jackson ImmunoResearch).

### Protocols for biomarker discovery analysis in prostate cancer

#### Prostate cancer plasma samples

Two different plasma sample collections from individuals with prostate cancer were profiled: The collection denoted as Study Set 1 consisted of a total of 90 EDTA plasma samples originating from a population-based case-control study. Here, the Study Set 1 cohort was divided into two main groups (T0/T1 or T3/T4) based on the tumor stage. Information about these sample groups is summarized in ***Table S2A***. The second sample collection, denoted here as Study Set 2, consisted of a total of 84 heparinized plasma samples donated by anonymized individuals with prostate cancer. Information about the sample groups is summarized in ***Table S2B***, where the individuals were divided into four groups: High total PSA (tPSA), low tPSA, elevated tPSA and low free-to-total PSA ratio (f/tPSA) and elevated tPSA and high (f/tPSA) based on the tPSA and f/tPSA values determined during clinical routine analysis. Further information on both of the sample collections, including the ethical approval, is provided elsewhere [2-4].

#### Direct labeling and single-capture assay of prostate cancer samples

Plasma samples were centrifuged for 10 min at 3,000 x g and aliquoted into microtiter plates with a liquid handling system (Freedom EVO150, TECAN). 3 μl of each sample was diluted in 22 μl of 1xPBS. The diluted and randomized samples were labeled utilizing biotin as previously described [1] Briefly, the diluted samples were incubated over 2 h at 4 °C with a 10-fold molar excess of NHS-PEG_4_-Biotin (Pierce) calculated based on the assumption of an average molecular mass of 60 kDa and a plasma/serum total protein concentration of 60 mg/ml. The labeling reaction was quenched by addition of a 250-fold molar excess of 0.5 M Tris-HCl (pH 8.0) over biotin. After incubation with Tris-HCl for 20 min at 4 °C, samples were stored back to -80 °C until usage.

The biotinylated samples were diluted 1:50 using a liquid handler (SELMA, CyBio) in an assay buffer composed of 0.5% (w/v) polyvinylalcohol and 0.8% (w/v) polyvinylpyrrolidone (Sigma) in 0.1% (w/v) casein (Sigma-Aldrich) in PBS (PVXC) supplemented with 0.5 mg/ml rabbit IgG (Bethyl). Samples were then heat-treated at 56 °C for 30 min and cooled to 20°C for 15 min in a thermo-cycler (Applied Biosystems). Then, 45 μl of heat-treated samples were added to 5 μl of the antibody suspension bead array distributed into a 96-well microtiter plate (Greiner BioOne), where approximately 500 beads per bead ID were combined. Subsequent to 16 h incubation on a shaker (Grant) at RT, beads were washed with 3 × 50 μl PBS-T using a plate washer (EL406, Biotek), followed by an incubation for 10 min with 50 μl of 0.4% paraformaldehyde in PBS-T. Beads were washed with 50 μl PBS-T and incubated with 50 μl of 0.5 μg/ml R-Phycoerythrin labeled streptavidin (Invitrogen) in PBS-T for 20 min. Finally, beads were washed 3 × 50 μl PBS-T before addition of 60 μl of PBS-T for measurement in the FlexMap3D instrument (Luminex Corp.) utilizing the Luminex xPONENT software. At least 50 events per bead ID were counted and binding events were displayed as median fluorescence intensity (MFI).

#### Dual-capture assay of prostate cancer samples

Plasma samples were centrifuged for 10 min at 3,000 x g and 10 μl were diluted in 20 μl assay buffer composed of 0.5% (w/v) polyvinylalcohol, 0.8% (w/v) polyvinylpyrrolidone (Sigma) and 0.1% (w/v) casein (Sigma-Aldrich) in 1xPBS supplemented with 0.05% Tween20 and 0.5 mg/ml rabbit IgG (Bethyl) using a liquid handler (SELMA, CyBio). The diluted samples were incubated over 2 h at 4 °C with 5 μl of first capture antibody bead array, approximately 1250 beads per bead identity were distributed in each well in a 96-well Thermofast PCR plate (AB-1300, Thermo Scientific ABgene). Then, beads were washed with 3 × 100 μl PBS-T (1×PBS, 0.05% Tween20) using a flex magnetic particle processor (KingFisher Flex, Thermo Scientific), followed by an incubation over 1 h at 4 °C with 30 μl of 300-fold molar excess of NHS-PEG_4_-Biotin (Pierce) diluted in PBS-Triton (1×PBS, 0.005% Triton-X100).

The labeling reaction was terminated by washing 3 × 100 μl PBS-T followed by elution of captured proteins by incubation with 15 μl of elution buffer 1 (0,1M Glycine-NaOH, 0,05% Tween20, pH 10) at 56 °C (water bath TW8, Julabo) for 20 min and cooled to RT for 10 min. Beads were thereafter transferred to 15 μl elution buffer 2 (2.5% acetic acid, 0.05% Tween20, pH 3), incubated at 56 °C for 20 min and cooled to RT for 10 min. Removal of beads was followed by a combination of the two elution buffers and neutralization at RT for 40 min by adding 30 μl neutralization buffer (pH 7.95) composed of 2M Tris, 0.2% (w/v) casein (Sigma-Aldrich), 1.0 % (w/v) polyvinylalcohol and 1.6% (w/v) polyvinylpyrrolidone (Sigma), in 1xPBS, supplemented with 0.5 mg/ml rabbit IgG (Bethyl), using a liquid handler (SELMA, CyBio). Five μl of the second capture antibody bead array, approximately 500 beads per bead identity were distributed into a 96-well microtiter plate (Greiner BioOne) and incubated with 50 μl neutralized protein eluate over 16 h on a shaker (Grant) at RT. Beads were washed with 3 × 100 μl PBS-T using a plate washer (EL406, Biotek), followed by an incubation for 10 min with 50 μl of 0.4% paraformaldehyde in PBS-T. Then, washed 3 × 100 μl PBS-T and incubated with 50 μl of 0.5 μg/ml R-Phycoerythrin labeled streptavidin (Invitrogen) in PBS-T for 30 min. Lastly, beads were washed 3 × 100 μl PBS-T before addition of 60 μl of PBS-T and measurement in the FlexMap3D instrument (Luminex Corp.). For each sample and bead ID, 50 events were collected and protein content was reported as median fluorescence intensity (MFI) and used for data analysis.

### Protocols for spike-in experiments with protein standards and plasma dilution series

#### Direct labeling and single-capture assay with protein standards or plasma dilution series

The protocol for direct labeling and single-binder assay described above was applied for analysis of various spike-in protein standards into 0.6-1 mg/ml BSA or a plasma pool. Likewise, dilution series of a plasma pool were analyzed as described above.

#### Dual capture assay with protein standards or plasma dilution series

The protocol for dual-capture assay described above was applied for analysis of various spike-in protein standards into 0.6-1 mg/ml BSA or a plasma pool. Likewise, dilution series of a plasma pool were analyzed as described above.

#### Protocol for sandwich assay on protein standards

Recombinant protein standards from the following R&D Systems DuoSet ELISA kits; IL-6 (DY206), IL-7 (DY207), F3 (DY2339), IL-5 (DY205), TNF-α (DY210) and VEGF (DY293B) were diluted in 1xPBS constituted with 1 mg/ml BSA (Sigma) and 0.06% Tween20. 3.3 μl of the protein standards were combined into six pools, each with one of the proteins absent, to a total volume of 165 μl and 3-fold diluted into a series of eight points. Thirty μl of each dilution were incubated for 2 h at RT on a shaker (Grant) with 5 μl of a 7-plex antibody bead array consisting of the corresponding DuoSet capture antibodies and an additional control bead without any antibody, approximately 500 beads per bead identity were distributed in each well in a 96-well microtiter plate (Greiner BioOne). Beads were washed 3 × 100 μl with 1xPBS 0.05% Tween20 (PBS-T) using a plate washer (EL406, Biotek), followed by an incubation with 30 μl of a DuoSet detection antibody cocktail (IL-6 0.1μg/ml, IL-7 0.5μg/ml, F3 0.5μg/ml, IL-5 0.5μg/ml, TNF-α 0.5μg/ml, VEGF 0.5μg/ml) for 1 h on a shaker at RT. Then, beads were washed with 3 × 100 μl PBS-T and incubated for 10 min with 50 μl of 0.4% paraformaldehyde in PBS-T followed by 3 × 100 μl PBS-T washing and incubation with 50 μl of 0.5 μg/ml R-Phycoerythrin labeled streptavidin (Invitrogen) in PBS-T for 30 min. Finally, beads were washed 3 × 100 μl PBS-T and 60 μl of PBS-T was added for FlexMap3D (Luminex Corp.) measurement. For each sample and bead ID, 50 events were collected and protein content was reported as median fluorescence intensity (MFI).

### Protocol for assay read-out by RCA

#### Streptavidin oligonucleotide conjugation

The sequences of all oligonucleotides used in this assay are listed in ***Table S4***. Streptavidin was conjugated to an amine-modified oligonucleotide (Integrated DNA Technologies [IDT]) using the Lightning-Link Streptavidin Conjugation Kit (Innova Biosciences). In brief, 2 µl of LL-Modifier reagent were added to 1.8 nmol of the amine-modified oligonucleotide dissolved in 10 mM sodium phosphate buffer (pH 8). The oligonucleotide solution was added directly onto the lyophilized streptavidin and incubated overnight at room temperature. The reaction was stopped by adding 2 µl of LL-quencher reagent.

#### Padlock probe phosphorylation

Padlock probes were phosphorylated with 0.1 U/μl of T4 Polynucleotide Kinase (Thermo Scientific) in supplied buffer A (50 mM Tris-HCl, 10 mM MgCl_2_, 5 mM DTT, 0.1 mM spermidine) and 1 mM ATP at 37 °C for 30 min, followed by inactivation of the enzyme at 75 °C for 10 min.

#### Signal amplification by RCA

Following the dual capture, beads were washed in PBS-T before coupling the streptavidin conjugated oligonucleotide to the biotinylated proteins. Fifteen µl of 2.5 nM oligonucleotide in PBS-T were added to the beads and incubated for 10 min at RT followed by two PBS-T washes. Padlock probes were hybridized and ligated onto the streptavidin conjugated oligonucleotide by adding 20 µl of a ligation mix (100 nM padlock probe, 0.2 mg/ml BSA [New England Biolabs], 1x T4 ligation buffer [66 mM Tris-HCl, 10 mM DTT, 10 mM MgCl_2_][DNA Gdansk], 50 mU/µl T4 DNA ligase [DNA Gdansk] and 0.7 mM ATP [DNA Gdansk]). Samples were incubated for 15 min at RT. Excess padlock probes were removed by applying one PBS-T wash. Ligated padlock probes were amplified by adding 20 µl of an RCA mix and incubating for 1 h at 37 °C followed by inactivation at 65 °C for 1 min. Rolling circle products were labeled by adding 20 µl of 0.01 µM detection oligo, 40 mM EDTA, 40 mM Tris-HCl (pH 8), 0.2% Tween-20, and 2 M NaCl. Samples were incubated for 2 min at 70 °C followed by 15 min at 55 °C. Finally, beads were washed 3x in PBS-T and 60 µl were added for analysis in Luminex instrument (Luminex Corp.).

### Data analysis and statistics

All data analysis and visualizations were performed using R. Dilution series were performed in triplicates and the lower limit of LOD was calculated based on a five-parametric logistic regression. We used log2-transformed mean MFI values and determined the LOD as 3× SD of the MFI value for the non-spiked reference sample (background) in each analysis. For analysis of data from the prostate cancer collection, MFI values were pre-processed using probabilistic quotient normalization (PQN) [5] accounting for any potential sample dilution effects and the PQN-normalized data was used in the statistical analyses displayed in the figures and tables. Principal component analysis (PCA) was performed to confirm that there was no systematic variation in the dataset and that there were no outlier samples.

# Supplementary Figures & Tables

### Supplementary Fig. 1 – Comparison of LOD values in DCA, single-capture assay and sandwich assay

**(A)** Six protein standards for IL-5, IL-6, IL-7, F3, TNF-α and VEGF were used to make six different pools where each standard was excluded from one of the pools as shown in the scheme. **(B)** A seven-point and three-fold dilution series was prepared in 0.6 mg/ml BSA for each pool, where the final concentration of each standard is summarized in the table. **(C-E)** The prepared dilution series for each pool was divided into three and each aliquot was analyzed in triplicate and in parallel, either in a single capture, dual capture or sandwich assay format. The tables summarize the lower LOD’s calculated for each standard in each pool analyzed in three different assay formats, as well as the average LOD, SD of LOD and %CV of LOD for each standard across the five pools. The ratio of average LOD in DCA with respect to average LOD in single-capture or sandwich assay is displayed at the bottom left and right, respectively.

###

### Supplementary Fig. 2 – Effect of detergent added during the labeling step on limit of detection in DCA

A seven-point and ten-fold dilution series of CA3 protein was prepared in 0.6 mg/ml BSA and divided into three aliquots. Following the first capture, the labeling solution for each aliquot was supplemented either with 0.05% Tween-20, 0.005% Triton-X or with no detergent. The y-axis displays the MFI values obtained for the anti-CA3 antibody and the x-axis displays concentrations of the spiked CA3 in ng/ml. All measurements were performed in triplicates. Error bars indicate SD. LOD for each labeling condition is indicated as inset.

###

### Supplementary Fig. 3 – Effect of high or low pH during elution on limit of detection in DCA

A seven-point and ten-fold dilution series of CA3 protein was prepared in 0.6 mg/ml BSA and divided into two aliquots. Following the steps of first capture and labeling on beads, captured CA3 was eluted either by 2.5% acetic acid, pH 3.0 or by 0.1M glycine-NAOH, pH 10.0. The y-axis displays the MFI values obtained for the anti-CA3 antibody and the x-axis displays concentrations of the spiked CA3 in ng/ml. All measurements were performed in triplicates. Error bars indicate SD. LOD for each elution condition is indicated as inset.

###

### Supplementary Fig. 4 – Comparison of pH conditions during elution on overall protein profiles in plasma

A pool of Study Set 1 plasma samples at a dilution rate of 1:3, as well as sample-free assay buffer was analyzed in triplicates, where four different elution conditions sequentially using 2.5% acetic acid, pH 3.0 and/or 0.1M glycine-NAOH, pH 10.0 were tested. **(A-B)** The line plots display the average MFI values obtained for the triplicate of each elution condition in the plasma sample and the blank over all antibodies in the 52-plex bead array. **(C)** SOTA was applied on scaled and centered MFI values across the four different pH combinations for an unsupervised and divisive clustering of protein profiles for the 52-plex bead array.

###

### Supplementary Fig. 5 – Selectivity in DCA compared to single capture assay

A ten-fold dilution series of recombinant CA3 protein spiked into 0.6 mg/ml BSA was analyzed with the 52-plex antibody array both in a single- and dual-capture assay format. The term “Blank” refers to the measurements that did not contain any spiked protein. The y-axes display the MFI values obtained for a small subset of the 52 antibodies, including an anti-CA3 antibody. All measurements were performed in triplicates. Error bars indicate SD.

###

### Supplementary Fig. 6 – Effect of number of beads at first capture

**(A-B)** A pool of Study Set 1 plasma samples at a dilution rate of 1:30, as well as sample-free assay buffer was analyzed in triplicates, where for the first capture step only a single bead population coupled to an anti-CNDP1 antibody or to no antibody was utilized. Three different total amount of beads, 2,000, 4,000 and 8,000, were used. Boxplots display all MFI values for the triplicates across all antibodies in the 52-plex bead array.


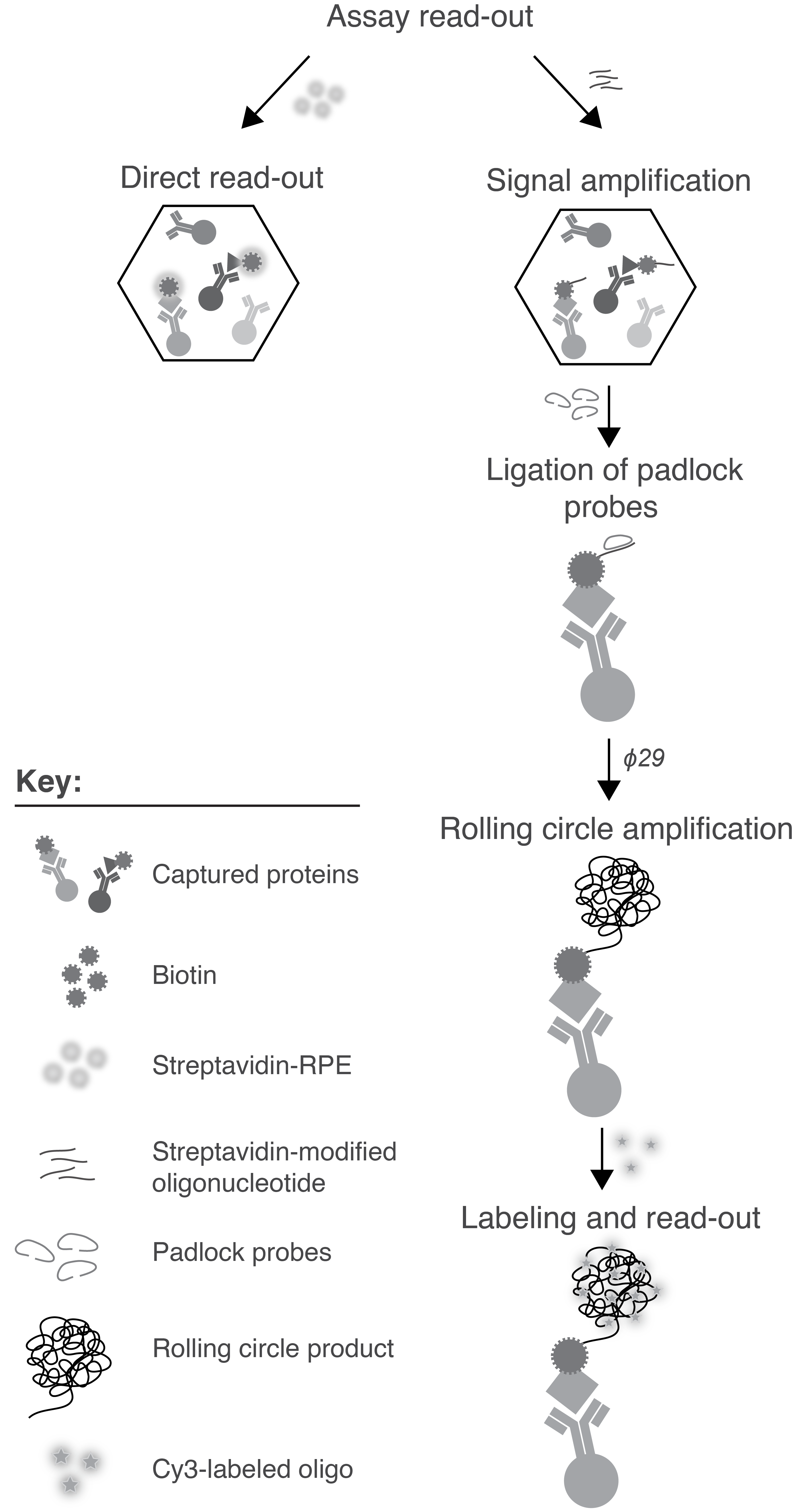


### Supplementary Fig. 7 – Assay read-out with rolling circle amplification

Captured, biotinylated proteins can be either directly labeled by SAPE or alternatively the signal can be amplified by RCA. For signal amplification, padlock probes are hybridized and ligated to a streptavidin-conjugated oligonucleotide, which is coupled to the biotinylated protein. Circularized padlock probes are amplified using RCA and the resulting rolling circle products are labeled by hybridizing short, fluorescently-labeled oligonucleotides.


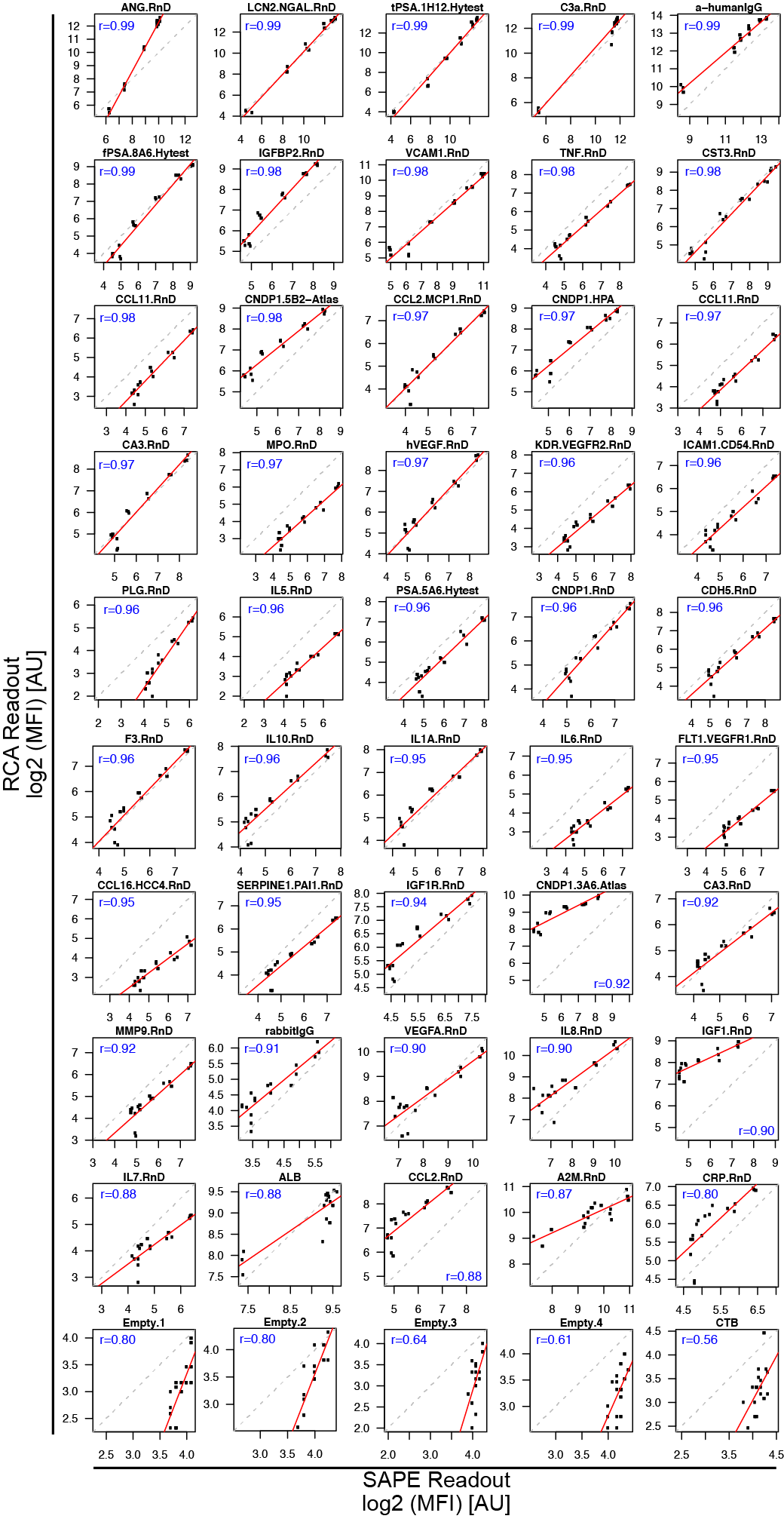


### Supplementary Fig. 8 – Effect of RCA on overall MFI values

A dilution series of a pool of Study Set 1 plasma samples was prepared at a four-fold dilution from 1:2 to 1:512 and aside of a sample-free buffer. The samples were divided into two aliquots, and both aliquots were analyzed in parallel as triplicates using the DCA format. For one aliquot, the signal generation occurred via direct read-out with SAPE, and the other aliquot was subjected to the RCA protocol for assay readout. The plots compare the MFI values obtained for each antibody across the plasma dilution series obtained via RCA readout (y-axis) and SAPE readout (x-axis). The red lines indicate the linear trends of the correlation, and Pearson’s correlation values are stated in blue. The dashed diagonal line represents the line of identity. Trend lines above the line of identity indicate higher MFI values for RCA detection, while trend lines below represent higher MFI values for SAPE detection. Generally, a high concordance was observed between RCA and SAPE detection. Background levels obtained from “Empty” beads (bottom row) were more consistent over the range of plasma dilutions when using SAPE detection. The levels obtained from RCA detection were however lower and showed to increase along with an increasing sample concentration.

### Supplementary Fig. 9 – CVs in SAPE- and RCA-based readout.

A dilution series of a pool of Study Set 1 plasma samples was prepared at a four-fold dilution from 1:2 to 1:512 and aside of a sample-free buffer. The samples were divided into two aliquots, and both aliquots were analyzed in parallel as triplicates using the DCA format. For one aliquot, the signal generation occurred via direct read-out with SAPE (blue), and the other aliquot was subjected to the RCA protocol (black) for assay readout. The plot compares the CVs in MFI values over the triplicates of each dilution point for each antibody.


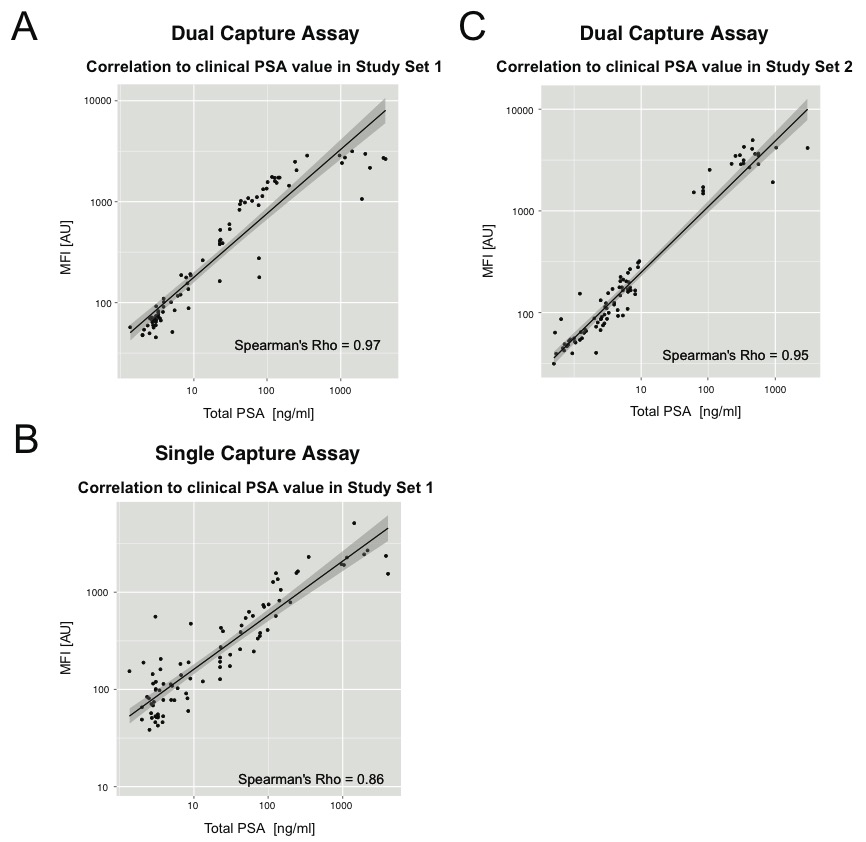


### Supplementary Fig. 10 – Correlation to clinical PSA values in Study Set 1 and 2

The y-axes in the scatterplots display the MFI values for the anti-total PSA antibody and the x-axes display the total PSA concentration determined in the clinic for all of the samples within Study Set 1 and 2. For Study Set 1, scatterplots including only those samples with clinical PSA values lower than 1 μg/ml are shown in ***Figure 3 A-B***.

###
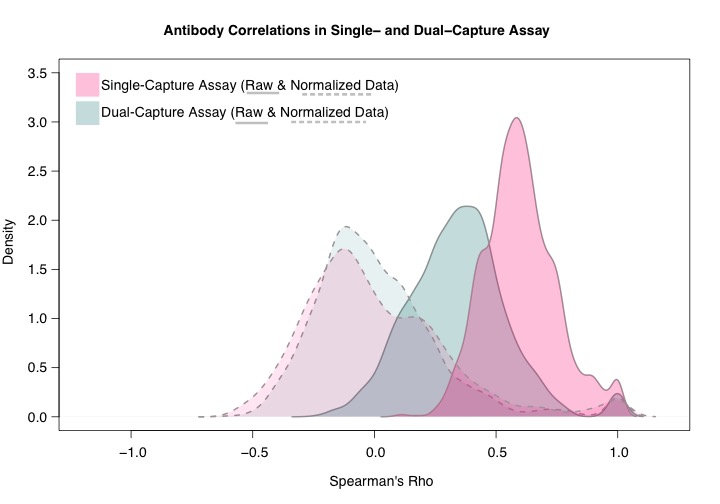


### Supplementary Fig. 11 – Distribution of antibody correlation coefficients in DCA and single-capture assay

Study Set 1 was analyzed using both a single- and a dual-capture assay format and the MFI values for each antibody across all samples were subjected to a pair-wise correlation using. The density plot displays the distribution of Spearman’s Rho correlation coefficient across all antibodies in the 52-plex bead array, both for the raw and PQN-normalized data.

### Supplementary Tab. 1 – Information on the antibodies used in the study

###

### Supplementary Tab. 2 – Information on the two prostate cancer plasma sample collections

***A – Study Set 1***

| **Group** | **Nr. of subjects** | **tPSA [ng/ml]** |
| --- | --- | --- |
| T0/T1 | 41 | 198 (1.36 – 9.09) |
| T3 | 31 | 85.7 (22.9 – 4075) |
| T4 | 10 | 3.13 (13.2 – 2500) |

***B – Study Set 2***

| **Group** | **Nr. of subjects** | **tPSA [ng/ml]** | **f/tPSA** |
| --- | --- | --- | --- |
| High tPSA | 20 | 565 (61 – 3030) | N/A |
| Low tPSA | 23 | 0.9 (0.5 – 1.5) | N/A |
| Elevated tPSA and low f/tPSA | 21 | 5.5 (2.1 – 9.4) | ≤ 0.15 |
| Elevated tPSA and high f/tPSA | 20 | 4.1 (2.0 – 9.0) | ≥ 0.30 |

### Supplementary Tab. 3 – P-values for differences revealed in two different prostate cancer study sets

The nonparametric Wilcoxon rank‐sum and Kruskal–Wallis tests were applied to raw or PQN‐normalized data to calculate  p-values for two- or multi-group comparisons. Differences in protein profiles between compared groups were denoted statistically significant for p-value  < 0.05 without multiple testing correction (highlighted in pink) or for p-values passing multiple testing correction threshold (p-value  < 0.0001, highlighted in green).

### Supplementary Tab. 4 – Sequence of the oligonucleotides used in RCA

| **Oligo Name** | **Sequence** |
| --- | --- |
| Amine-modified | 5'-Amino modifier C6-CTCTCTCTCT CTCTCTCTCT TACGATGGTC GATCTGGCTT-3’ |
| Padlock probe | 5’-GACCATCGTA GTGTATGCAG CTCCTCAGTA TAGTCGATAG TCACGGCTAC TAAGCCAGAT C-3’ |
| Detection | 5’-Cy3-AGTCGATAGTCACGGCTACTTTTCmUmUmUmU-3’ |

## References

[1] Schwenk, J. M., Gry, M., Rimini, R., Uhlén, M., Nilsson, P., Antibody suspension bead arrays within serum proteomics. *J Proteome Res* 2008, *7*, 3168-3179.

[2] Schwenk, J. M., Igel, U., Neiman, M., Langen, H.*, et al.*, Toward next generation plasma profiling via heat-induced epitope retrieval and array-based assays. *Mol Cell Proteomics* 2010, *9*, 2497-2507.

[3] Qundos, U., Johannesson, H., Fredolini, C., O’Hurley, G.*, et al.*, Analysis of plasma from prostate cancer patients links decreased carnosine dipeptidase 1 levels to lymph node metastasis. *Translational Proteomics* 2014, *2*, 14-24.

[4] Nordstrom, M., Wingren, C., Rose, C., Bjartell, A.*, et al.*, Identification of plasma protein profiles associated with risk groups of prostate cancer patients. *Proteomics Clin Appl* 2014, *8*, 951-962.

[5] Dieterle, F., Ross, A., Schlotterbeck, G., Senn, H., Probabilistic quotient normalization as robust method to account for dilution of complex biological mixtures. Application in 1H NMR metabonomics. *Anal Chem* 2006, *78*, 4281-4290.
